# Applying KT Network Complexity to a Highly-Partnered Knowledge Transfer Effort: Comment on "Using Complexity and Network Concepts to Inform Healthcare Knowledge Translation"

**DOI:** 10.15171/ijhpm.2017.141

**Published:** 2017-12-17

**Authors:** JoAnn E. Kirchner, Sara J. Landes, Aaron E. Eagan

**Affiliations:** ^1^QUERI for Team-Based Behavioral Healthcare, Central Arkansas Veterans Healthcare System, Little Rock, AR, USA.; ^2^VISN 16 South Central Mental Illness Research Education and Clinical Center (MIRECC), Central Arkansas VA Health Care System, Little Rock, AR, USA.; ^3^Office of Mental Health and Suicide Prevention, Department of Veterans Affairs, Gainesville, FL, USA.

**Keywords:** Knowledge Translation, Complexity Theory, Implementation Science, Healthcare, Partnered Research

## Abstract

The re-conceptualization of knowledge translation (KT) in Kitson and colleagues’ manuscript "Using Complexity and Network Concepts to Inform Healthcare Knowledge Translation" is an advancement in how one can incorporate implementation into the KT process. Kitson notes that "the challenge is to explain how it might help in the healthcare policy, practice, and research communities." We propose that these concepts are well presented when considering highly-partnered research that includes all sectors. In this manuscript we provide an example of highly-partnered KT effort framed within the KT Complexity Network Theory. This effort is described by identifying the activities and sectors involved.

The re-conceptualization of knowledge translation (KT) in Kitson and colleagues’ manuscript^1^ “Using Complexity and Network Concepts to Inform Healthcare Knowledge Translation” is an advancement in how one can incorporate implementation into the KT process. First, some may consider the implementation of an innovation to be synonymous with KT. In this manuscript, how these processes differ is clearly demonstrated. Figure 3 of the Kitson manuscript provides a clear depiction of the processes involved in KT (including implementation) as well as the five sectors that can independently or jointly influence KT. These sectors - *community, health [systems], government, education, and research* - encompass the primary sources of influence. Second, this manuscript posits that KT is neither linear nor cyclical, as is frequently depicted. Rather how KT occurs is dependent upon the interaction of the KT processes and five sectors. Kitson describes the processes of KT as *problem identification*, *knowledge synthesis, implementation*, *evaluation,* and *knowledge creation.* Third, the induction of complexity theory into KT supports the proposition that outcomes of KT processes are often “unpredictable, non-linear, and emergent.” For those of us deeply involved in KT activities as well as its study, this is quite reassuring.

Kitson notes that “the challenge is to explain how it might help in the healthcare policy, practice, and research communities.” These concepts were very useful to us, as they capture the unpredictable and constantly evolving nature of KT and research that is highly-partnered between the sectors described by Kitson. The purpose of this commentary is to provide an example of an ongoing highly-partnered KT effort framed within the KT Complexity Network Theory and describe how the KT model can be used to elucidate factors that impact the KT effort.

This example is taken from activities conducted within the United States Department of Veterans Affairs (VA). Of note, this example is work that is actively under way and subject to further changes in the months and years to come, as expected according to the KT model. Applying the KT model during the course of a project or study allows participants to use the model to understand and address challenges that emerge from different sectors during the course of the knowledge transfer. The *problem identified* is military Veteran suicide and the need to identify those at highest risk for suicide to improve suicide prevention efforts. To address this problem VA* created* a predictive model of suicide risk using existing electronic medical record data,^2,3^ a dashboard to inform providers of high risk Veterans, and a suicide prevention clinical initiative (review, enhancement, and outreach) using this information. Information was* synthesized* into initiative resources that include: educational handouts, video examples, medical record note templates, scripts to use when discussing suicide prevention with Veterans, and other tools. The *implementation* included policy memos that encompass the national VA healthcare system, identification of a coordinator at each of the 140 VA healthcare systems, web-based training, educational and support materials, and technical assistance. For sites having difficulty fully implementing the program external implementation facilitation^4,5^ is provided. The *evaluation* includes three components: the evaluation of patient outcomes and effectiveness of the suicide prevention clinical intervention, evaluation of program implementation and use of implementation facilitation, and an iterative adaptation of the dashboard and other tools based on feedback from community and healthcare stakeholders.

The five sectors (or stakeholders) include *the Community*: Veterans, family members, and other community stakeholders*; the Health System*: the United States Department of Veterans Affairs Veteran Healthcare Administration*; Education*: a clinical implementation team that focuses on educating providers, facility coordinators, and other stakeholders;* Government*: leadership of VA; and *Research*: an arm of VA that supports the evaluation described above. In this case, there is an overlap between the health system and the government, as the VA is a government agency with the mission of providing care to the nation’s military Veterans. This is likely similar to other government funded healthcare systems across the world. It is also likely different from other healthcare organizations that may be impacted by the government, but are not funded by the government, as is common in the United States. This research was supported through a Learning Healthcare Initiative randomized program evaluation that required early interactions between the research team, education specialists, the healthcare partner, and community stakeholders (See Figure).

**Figure:**
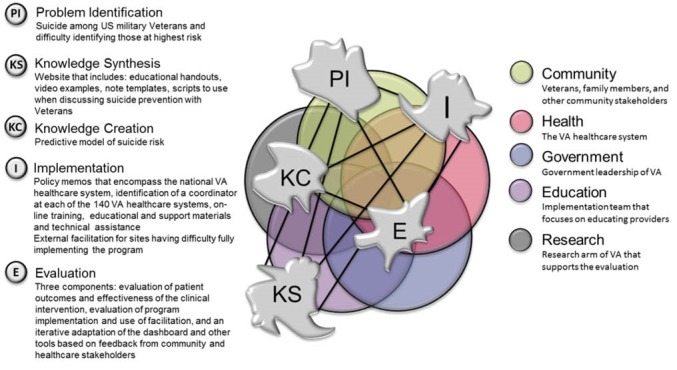
Applying the KT Complexity Network to a Healthcare Example. Abbreviation: KT, knowledge translation.

At the conclusion of their article, Kitson et al ask readers to consider how to facilitate networks and support nodes to flourish across complex adaptive systems and how to recognize and use the energy within systems rather than fight it. We believe that the VA’s new Learning Healthcare Initiative funding does just this by providing a way for operational offices (eg, *health system* and *government*) to partner with *research* colleagues to evaluate healthcare initiatives. This allows the health system to learn and improve, while at the same time advancing research on such KT activities. This works with this energy, as opposed to researcher initiated projects that are well-designed ways to test research questions, but might not fit the current healthcare and government priorities. Given that another mission of the VA is to provide *education*, there are built in collaborators with whom to help develop educational materials to support health system and government in KT activities. Finally, a core value of VA is to include the Veteran voice and most initiatives strive to include *community* stakeholders to inform their work.

As described in the Kitson article, KT is not occurring in a linear nor cyclical manner. Instead, interaction of the different sectors directly impacts the processes and the order in which they occur. The Learning Healthcare Initiative provided 6 months of integrated planning during which *research*, *healthcare*, *education,* and *community stakeholder* sectors worked together to synthesize knowledge around the clinical intervention and develop the implementation and evaluation plan. Yet, while this planning resulted in a randomized design through which implementation and evaluation would occur in a stepped wedge design over the course of four years, attention by *government* leadership to Veteran suicide accelerated the timeline to have system-wide implementation over a 3-month period. This, in turn, dictated a change in the implementation plan and evaluation by the *research*, *education*, and *healthcare* sectors. Since the intervention was implemented at one time point system-wide, the ability to test, evaluate, and iteratively modify clinical responses to the identification of Veterans at risk for suicide was compromised. Therefore, *research* and *healthcare* stakeholders modified the evaluation of these clinical interventions to a pre-post design as opposed to a controlled design.^6^

Healthcare facilities initially targeted for implementation facilitation assistance were identified during the planning stage as those in need of additional support to successfully implement the initiative. To enhance efficiency of the facilitation team and control for regional characteristics, *the research, education,* and *healthcare* stakeholders determined that facilitation would occur by region; seven regions (and the four lower performing facilities within the region) would receive facilitation in a stepped wedge design. This resulted in only seven units to be randomized, which rendered randomization inappropriate. Therefore, the research team used information about the facilities and regions to assign each to a start time for facilitation. The facilities within the region that needed assistance changed over the course of initial KT. Because this was a highly-partnered research initiative the *research* team adapted their model to include those sites of highest need of assistance and, fortunately, the changes would not have resulted in a change of order.

While the application of the KT model has allowed us to better understand the “chaos” that is occurring during this highly-partnered effort and channel the emerging energy, there are limitations to the application of the model. Detailing the processes and contexts in which knowledge transfer plays out well or fails to do so is not incorporated into the KT model. Thus, this is left to the *research* sector to incorporate these critical aspects of knowledge transfer in the *evaluation* of the *implementation*. In addition, not all *processes* are distinct. For example, products developed in our *knowledge synthesis* process were used in *implementation*. We suspect that this would be true of most KT efforts. Kitson also challenges those applying the KT Model to determine how they would know that they “know you have all the key stakeholders around the table at the same stage?” In our experience this did not occur. Rather, stakeholders vary in their presence and role across the course of the KT effort. This dynamic and changing stakeholder presence is one challenge that the KT model helps us better understand. Finally, while VA is making purposeful steps to advance partnered research, not all KT efforts need to be partnered to lead to positive outcomes and even partnered research can lack benefit to the organization in which it occurs. Again, this further enforces the need for partnered research activities to be comprehensively documented and their outcomes, beneficial or not, reported to all stakeholders as well as the broader KT community.

This highly-partnered knowledge transfer effort is currently in its early stages and we anticipate that the five sectors will continue to influence the processes that occur. While this may be challenging to those trained in traditional research methodology, as noted by Kitson, engagement of the sectors is vital for connections to take place. This is the challenge and the gift for those of us engaged in the study and conduct of highly-partnered KT.

## Acknowledgements

This article was supported by the Team Based Behavioral Health Quality Enhancement Research Initiative (QUERI) grant (QUE 15-289) and the Risk Stratified Enhancements to Clinical Care: Targeting Care for Patients Identified Through Predictive Modeling as Being at High Risk for Suicide, with the Office of Mental Health Operations grant (SDR 16-195) through the US Department of Veteran Affairs.

## Ethical issues

Not applicable.

## Competing interests

The views expressed in this paper are those of the authors and do not necessarily reflect the position or policy of the United States Department of Veterans Affairs (VA), Veterans Health Administration (VHA), or the United States Government.

## Authors’ contributions

JEK and SJL were responsible for the conception of the commentary and drafted the article. AEE provided critical revision of the article. All authors provided final approval of the version to be published.

## Authors’ affiliations

^1^QUERI for Team-Based Behavioral Healthcare, Central Arkansas Veterans Healthcare System, Little Rock, AR, USA. ^2^VISN 16 South Central Mental Illness Research Education and Clinical Center (MIRECC), Central Arkansas VA Health Care System, Little Rock, AR, USA. ^3^Office of Mental Health and Suicide Prevention, Department of Veterans Affairs, Gainesville, FL, USA.
